# Hydrothermal Cation Exchange Enabled Gradual Evolution of Au@ZnS–AgAuS Yolk–Shell Nanocrystals and Their Visible Light Photocatalytic Applications

**DOI:** 10.1002/advs.201700376

**Published:** 2017-11-20

**Authors:** Jingwen Feng, Jia Liu, Xiaoyan Cheng, Jiajia Liu, Meng Xu, Jiatao Zhang

**Affiliations:** ^1^ Beijing Key Laboratory of Construction‐Tailorable Advanced Functional Materials and Green Applications School of Materials Science & Engineering Beijing Institute of Technology Beijing 100081 China

**Keywords:** cation exchange, core‐shell, metal/semiconductor hetero‐nanocrystals, visible light photocatalysis, yolk–shell

## Abstract

Yolk–shell hybrid nanoparticles with noble metal core and programmed semiconductor shell composition may exhibit synergistic effects and tunable catalytic properties. In this work, the hydrothermal cation exchange synthesis of Au@ZnS–AgAuS yolk–shell nanocrystals (Y–S NCs) with well‐fabricated void size, grain‐boundary‐architectured ZnS–AgAuS shell and in situ generated Au cocatalyst are demonstrated. Starting from the novel cavity‐free Au@AgAuS core‐shell NCs, via aqueous cation exchange reaction with Zn^2+^, the gradual evolution with produced Au@ZnS–AgAuS Y–S NCs can be achieved successfully. This unprecedented evolution can be reasonably explained by cation exchange initialized chemical etching of Au core, followed by the diffusion through the shell to be AgAuS and then ZnS. By hydrothermal treatment provided optimal redox environment, Au ions in shell were partially reduced to be Au NCs on the surface. The UV–vis absorption spectra evolution and visible light photocatalytic performances, including improved photodegradation behavior and photocatalytic hydrogen evolution activity, have demonstrated their potential applications. This new one‐pot way to get diverse heterointerfaces for better photoinduced electron/hole separation synergistically can be anticipated for more kinds of photocatalytic organic synthesis.

The design and fabrication of hierarchical hetero‐nanocrystals with programmed compositions and morphologies is a hot topic due to their applications in a variety of fields, such as drug delivery, nanocatalysis, energy storage and conversion.^1^ Yolk–shell nanocrystals (Y–S NCs) as a promising platform for constructing functional nanomaterial have drawn significant attention,^2^ partly owing to the existence of an interior void which can accommodate guest molecules and function as a catalytic nanoreactor.^3^ Several strategies have been demonstrated to be applicable for building up nanovoids in hybrid nanostructures, including the methods based on Kirkendall effect, galvanic exchange, chemical etching, and anion exchange.[[qv: 3a,4]] In addition to pursuing versatile tailorability and functionality in both the core and shell, the scientific effort has also been directed toward regulating the particle sizes, shapes, and compositions of the Y–S NCs. A wide range of combinations of Y–S NCs have been studied, including metal@silica,^5^ metal@metal sulfide,^6^ metal@metal oxide,^7^ metal oxide@silica Y–S structures and so on.^8^ Among which, metal@semiconductor hybrid Y–S NCs are particularly attractive to serve as nanoconfined catalyst and display synergistic properties arising from the interplay of core and shell components.^9^ For example, Au@TiO_2_,^10^ Au@Cu_2_O,^11^ Au nanorod@Cu_7_S_4_,^12^ Au@Cu_2_S,^13^ Pd@Cu_2_S,^14^ Y–S NCs have shown enhanced activities in several important thermal catalytic reactions.

Recently, the potential of Y–S NCs in photocatalysis applications have been recognized. Fu et al. demonstrated the feasibility of using Pt@CeO_2_ Y–S nanocomposite as a visible light photocatalyst in selective oxidation of alcohol.^15^ The fabrication of TiO_2_@TiO_2_, Fe_3_O_4_@TiO_2_, and Zn_2_SnO_4_/SnO_2_ Y–S structures with improved photocatalytic activities in Rhodamine B degradation have also been demonstrated.^16^ However, plasmonic metal@semiconductor hybrid Y–S NCs, which can display plasmon‐exciton coupling for enhancing photocatalytic performance, have been rarely explored in this area. Moreover, in many reported cases, the Y–S nanostructures feature a freely movable core without any intimate contact between the core and shell. From the aspect of photocatalytic application, the capability to construct heterointerfaces in a single unit of the Y–S architecture is highly favored for promoting photoinduced electron/hole separation and utilization. Also, the flexible control of the interior void between the plasmonic core and the semiconductor shell can provide an effective tool for tuning the plasmon‐exciton coupling properties. Developing a facile approach to simultaneously achieve the above mentioned effects is desirable but remains a significant challenge. ZnS, a direct wide band gap (3.7 eV) semiconductor, possesses a highly negative conduction band and a large driving force for photocatalytic H_2_ evolution from water. However, its potential as good photocatalysts is hampered by the limited ability for solar energy harvesting (UV‐responsive only) and fast recombination of photoexcited charge carriers. Noble metal deposition, ionic doping, and combination with narrow band gap semiconductors are expected as useful strategies for ameliorate the photocatalytic performance of ZnS in the visible light region.^17^

Keeping these challenges in mind, in this study, we established a hydrothermal cation exchange strategy to prepare Au@ZnS–AuAgS Y–S NCs with several distinct structural features. Through aqueous cation exchange and cation exchange‐facilitated gradual chemical etching and diffusion of Au core: the shape of the Au core and accordingly the interior void could be gradually modulated, while an intimate contact between the core and the shell is always fulfilled due to the anisotropic nature of the etching process; the shell domain is highly crystalline and concurrently encompasses the wide band gap ZnS and the narrow band gap AuAgS, together with the existence of metal (Au, Ag) ion doped ZnS; via kinetics control of the hydrothermal cation exchange process, the outward diffusion of Au ions from the core can in situ generate Au cocatalysts on the external surface of the shell. Thus, multiple heterointerfaces between plasmonic Au core‐semiconductor shell, ZnS–AgAuS in the semiconductor shell, and semiconductor shell‐Au cocatalyst can be created in a single particle of the Y–S NC. These structural features are propitious for harnessing the surface plasmon resonance effect and boosting photoinduced charge carriers separation and utilization, which well rationalized the exceptional performance of Au@ZnS–AuAgS Y–S NCs in photocatalytic H_2_ evolution under visible light irradiation.

Water‐dispersive Au NCs with a spherical morphology and the size of ≈35 nm (Figure S1, Supporting Information) were adopted as the core material for fabrication of Au@ZnS–AgAuS Y–S NCs. The first step was epitaxial growth of a thin Ag layer around the Au NCs with controlled thickness to provide a crucial platform for the next chemical transformation stage. The uniform Au@Ag core‐shell NCs were obtained, where a AgAu alloy transition layer may contribute to a considerable content of the shell because of the close lattice parameters of Ag and Au.^18^ Subsequently, due to the comparable electronegativity of silver and sulfur, an in situ conversion of the metallic shell into metal sulfide was readily achieved based on sulfuration reaction. This procedure lead to the formation of a crystalline AgAuS shell,^19^ as suggested by the result of X‐ray diffraction (XRD) characterization displayed in **Figure** **1**. Starting from the resultant Au@AgAuS core‐shell structure, cation exchange reaction between Au^+^, Ag^+^ in the shell with Zn^2+^ in aqueous solution was carried out under hydrothermal condition in autoclave at 120/160 °C. However, many cation exchange reactions are thermodynamically nonspontaneous because of Δ*G* reaction > 0, as shown in reaction (1). Considering that tributylphosphine (TBP) is an important ligand which can be used to stabilize a variety of transition metal complexes and induce different cooperation modes between M and N cations and promote reaction (1) (ΔG reaction < 0), trace amount of TBP was introduced into the system to alter the thermodynamic preference of the reactions (reaction (2)) based on the hard and soft acids and bases theory^20^

(1) $$\mathrm{nMxEm}\;\left(\mathrm{molecule}\right)+{\mathrm{mxN}}^{n+}=\mathrm{mNxEn}\;\left(\mathrm{molecule}\right)+{\mathrm{nxM}}^{m+}$$

(2) $$\begin{array}{l} \mathrm{Zn}{\left({\mathrm{NO}}_{3}\right)}_{2}+6{\left(C_{4}H_{9}\right)}_{3}P+\mathrm{AgAuS}\;\left(\mathrm{NCs}\right)\to \mathrm{ZnS}\;\left(\mathrm{NCs}\right)\\ \;\;\;\;\;\;\;\;\;\;\;\;\;\;+{\left({\left(C_{4}H_{9}\right)}_{3}P\right)}_{3}\mathrm{Ag}-{\mathrm{NO}}_{3}+{\left({\left(C_{4}H_{9}\right)}_{3}P\right)}_{3}\mathrm{Au}-{\mathrm{NO}}_{3} \end{array}$$

**Figure 1:**
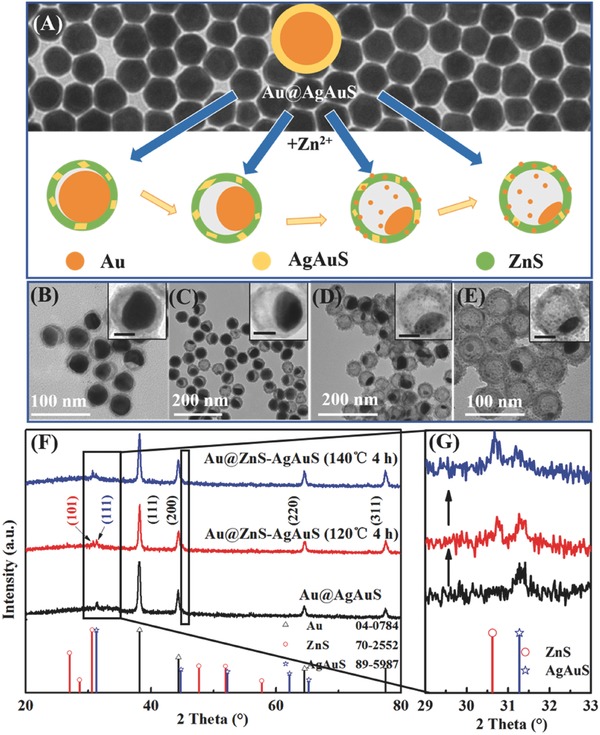
A) Schematic process for the preparation of Au@ZnS–AgAuS Y–S NCs. Background shows the TEM image of Au@AgAuS core‐shell NCs. B–E) TEM images showing in situ chemical conversion reaction kinetics dependent evolution of Au@ZnS–AgAuS Y–S NCs, which were obtained at (B) 120 °C for 2 h and denoted as Au@ZnS–AgAuS (120 °C 2 h), (C) 120 °C for 4 h, denoted as Au@ZnS–AgAuS (120 °C 4 h), (D) 140 °C for 4 h, denoted as Au@ZnS–AgAuS (140 °C 4 h), and (E) 160 °C for 4 h, denoted as Au@ZnS–AgAuS (160 °C 4 h), respectively. Insets show the corresponding TEM images of the individual Y–S NCs (scale‐bar 20 nm) with enlarging of interior voids. F) XRD patterns of Au@AgAuS core‐shell NCs, Au@ZnS–AgAuS (120 °C 4 h) and Au@ZnS–AgAuS (140 °C 4 h) Y–S NCs. G) Enlarged XRD patterns.

The morphology evolution of the resulting Y–S NCs versus the time and temperature of hydrothermal cation exchange was revealed by the large‐scale low‐resolution transmission electron microscope (LRTEM) images, as shown in Figure 1B–E (see also Figure S2 in the Supporting Information). The as‐prepared Y–S NCs exhibited a uniform particle size of about 50 nm, with a shell thickness of 5 nm. The overall morphology of the cation exchange products was similar to the original Au@AgAuS core‐shell structures. However, an asymmetric hollow void was observed as the exchange reaction continued. At limited reaction time of cation exchange (≈30 min at 120 °C), the holonomic core‐shell structure was obtained (Figure S3, Supporting Information). With the hydrothermal temperature kept at 120 °C, the void started to appear after 2 h of hydrothermal cation exchange, and continuously expanded as the reaction time was prolonged to 4 h (Figure 1B,C). Interestingly, when the temperature was increased up to 140 and 160 °C, the void was further enlarged accompanied with the appearance of small Au NCs on the shell surface (Figure 1D,E). According to the XRD patterns of Au@ZnS–AgAuS (120 °C 4 h) and Au@ZnS–AgAuS (140 °C 4 h) Y–S NCs in Figure 1F, aside from the peaks corresponding to Au and AgAuS, a notable peak from wurtzite ZnS (JCPDS File No. 70‐2552) was observed after cation exchange with Zn^2+^, suggesting the coexistence of ZnS and AgAuS phases in the Y–S NCs. In addition, as shown in Figure 1G, the characteristic peak of ZnS exhibited a slight shift, which was probably attributed to the cation doping (such as Ag^+^, Au^+^) in ZnS matrix during the cation exchange reaction process. This is consistent with the findings of several research groups, such as Banin and co‐workers and Norris and co‐workers, who have demonstrated that cation exchange enabled efficient ion doping in semiconductor NCs.^21^ Furthermore, with increasing reaction temperature from 120 to 140 °C, the intensities of XRD peaks from ZnS were increased notably, indicating the enhanced crystallinity of ZnS and the changed composition of the semiconductor shell at higher temperatures.

For comparison, it is worth noting that when the hydrothermal treatment of Au@AgAuS core‐shell was carried out at 140 °C for 4 h without Zn^2+^ and TBP, Y–S nanostructures could not be obtained (Figure S4, Supporting Information), indicating the cation exchange process is a critical procedure for the controllable formation of interior voids. The produced Y–S NCs are very stable in solution, no considerable structural changes were observed over several months. By adjusting the time and temperature of the cation exchange reaction process, Au@ZnS–AgAuS Y–S nanostructures with tunable interior voids and compositions have been fabricated well here.

The morphological, structural, and compositional characterizations of Au@ZnS–AgAuS (120 °C 4 h) and Au@ZnS–AgAuS (140 °C 4 h) Y–S NCs are further discussed by the high‐resolution TEM (HRTEM) characterizations. The HRTEM images acquired from two conjoint regions in one Au@ZnS–AgAuS (120 °C 4 h) Y–S NC (**Figure** **2**A) are shown in Figure 2B, in which the indexed lattice spacing of 0.30 and 0.28 nm are assigned to the (101) planes of wurtzite ZnS and the (111) planes of cubic AgAuS, consistent with the XRD results (Figure 1F). The dark‐field scanning TEM (STEM) image in Figure 2C combined with energy dispersive spectroscopy (EDS) elemental mapping (Figure 2D) of an individual Au@ZnS–AgAuS (120 °C 4 h) NC clearly reveals the direct contact between Au core and ZnS–AgAuS shell. When it comes to the Au@ZnS–AgAuS (140 °C 4 h) Y–S NC in Figure 2G, three kinds of heterointerfaces are observed synergistically, including Au‐ZnS yolk–shell heterointerface, Au cocalalyst‐shell heterointerface, and ZnS–AgAuS grain boundaries (see from Figure 2E,F,H; Figure S5 in the Supporting Information).

**Figure 2:**
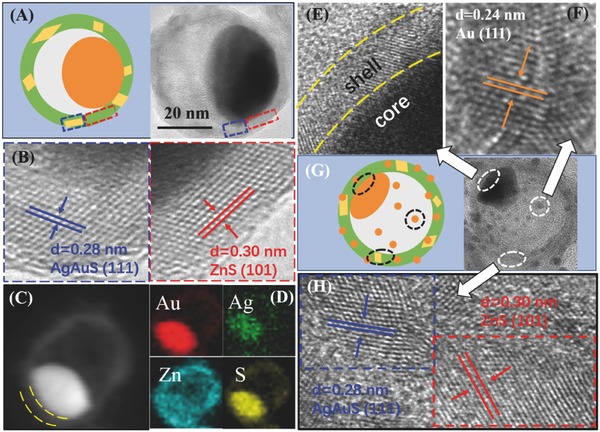
A) Diagram and TEM image of Au@ZnS–AgAuS (120 °C 4 h) Y–S NCs. B) HRTEM images acquired from two conjoint regions on the Au@ZnS–AgAuS NCs in (A). C) STEM image and D) STEM‐EDS elemental maps of Au@ZnS–AgAuS (120 °C 4 h) Y–S NCs. E–H) HRTEM images (E, F, H) and Diagram (G) of Au@ZnS–AgAuS (140 °C 4 h) Y–S NCs with three kinds of heterointerface synergistically.

The surface chemical states of Au@ZnS–AgAuS (140 °C 4 h) Y–S NCs were characterized by their X‐ray Photoelectron Spectroscopies (XPS). As shown in the XPS survey spectra in Figure S6 in the Supporting Information, Ag, Zn, Au, S, C, and O are detected from the products. The presence of O may attribute to the oxygen adsorption. The high‐resolution Au 4f spectrum shows binding energy peaks at 84.0, 84.9, and 88.4 eV, which can be assigned to Au^0^, Au^+^, and Au^3+^, respectively (**Figure** **3**A).^22^ Since Zn 3p also has a peak at around 89 eV, the asymmetric peak is the overlying of Zn and Au chemical state. The spectra of Ag 3d (Figure 3B) showed two symmetric peaks due to spin‐orbit splitting with binding energies of 373.8 and 367.8 eV for the d_3/2_ and d_5/2_ lines, indicating that Ag presents only the chemical state of Ag^+^. As shown in Figure 3C, two strong peaks at approximately 1045.3 and 1022.2 eV were assigned to Zn 2p_1/2_ and 2p_3/2_, respectively, which belongs to Zn^2+^. In Figure 3D, the peak of S 2p spectrum was deconvoluted into two components by Gaussian fitting, which can be assigned to the spin‐orbit splitting to S 2p. The peaks centered at 161.4 and 162.5 eV implied that S is in anion form, which agrees well with literature.^23^ The coexistence of Au^0^, Au^+^, and Au^3+^ has been carefully understood. Based on literature reports, cetyltrimethylammonium bromide (CTAB) together with H_2_O_2_ are usually used to tailor the resonance wavelength of Au nanorods through anisotropic shortening. Herein, hydrothermal cation exchange condition with dissolved oxygen (from air) provides a certain oxidizing environment for the oxidation of Br^−^ from CTAB to elemental bromine. As the spontaneous reaction between Au^0^ and Br_2_, Au core can be easily oxidized.^24^ The reaction is shown as the following

(3) $${\mathrm{Au}}^{0}+2{\mathrm{Br}}_{2}+e^{-}\to {\mathrm{AuBr}}_{4}{}^{-}\;\left(E^{0}=+0.236\right)$$

on the other hand, the bromide and bromine can rapidly bind together to form Br_3_^−^, which can act with Br^−^ and Au° following the equation^25^

(4) $$2{\mathrm{Au}}^{0}+{\mathrm{Br}}_{3}{}^{-}+{\mathrm{Br}}^{-}\to 2{\mathrm{AuBr}}_{2}{}^{-}$$

**Figure 3:**
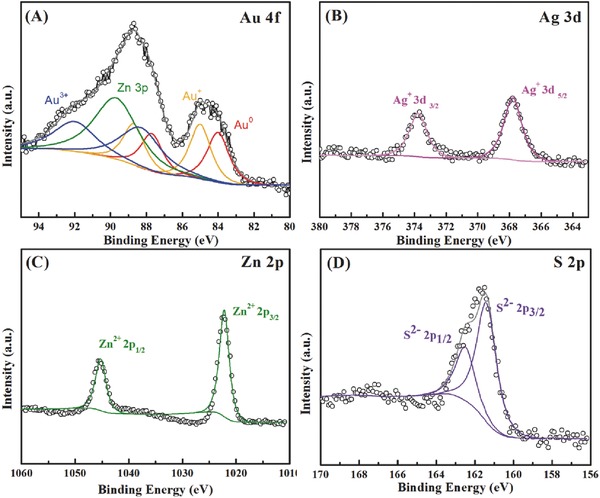
High resolution XPS spectra of A) Au 4f, the green line represents the peak from Zn 3p, B) Ag 3d, C) Zn 2p, and D) S 2p from Au@ZnS–AgAuS (140 °C 4 h) Y–S NCs.

In such way, Au cores are oxidized by bromine to be Au^+^ and Au^3+^. These conclusions are consistent with the XPS results in Figure 3. As a control experiment, the hydrothermal cation exchange reaction was carried out in the presence of hexadecyltrimethylammonium chloride (CTAC) and the same morphology of products can be observed (see Figure S7 in the Supporting Information), indicating the corrosion behavior of halide ions during the hydrothermal treatment process.

Combining all of the experimental observations together, the hollowing process is believed to be the cation exchange initialized chemical etching and diffusion of Au from the inner core to the outer shell. The hydrothermal treatment, Br^−^ and dissolved O_2_ provides a certain oxidative environment for Au oxidation to be Au ions then diffuse through the shell and turn into AgAuS. TBP and Zn^2+^ in the colloid suspension initiate the cation exchange reaction between Au^+^, Ag^+^ in shell and Zn^2+^ due to the strong coordination of Au^+^, Ag^+^ with TBP, resulting in the ZnS–AgAuS heterograin boundary formation. When the Au core is further corroded, the extra Au ions diffuse to the surface of the sulfide shell and capture electrons from the solution, which are finally in situ reduced to be Au NCs as cocatalysts.^26^ This kind of diffusion of Au from inside to outside occurred in many systems.^26^, ^27^ Yang and co‐workers observed the diffusion of gold core to Ag_2_S shell, and Wang and co‐workers reported the transformation from Au core‐sulfide shell to Au nanoparticle‐decorated sulfide hybrid nanostructures.[[qv: 26,27b]]

The optical properties of these Au@ZnS–AgAuS Y–S NCs show interesting tunability during the evolution process. The UV‐Vis spectra comparison of Au and as‐prepared Y–S NCs has been demonstrated. As shown in **Figure** **4**A, the spherical Au NCs demonstrate a sharp localized surface plasmon resonance (LSPR) peak at 530 nm, while the as‐synthesized Y–S NCs have stronger visible light absorption. Moreover, the LSPR peak in wavelength range from 570 to 617 nm can be observed in the case of Y–S NCs, which are sensitively dependent on the Au core size, shape, shell composition and the refractive index of the surrounding medium. These observations indicate the SPR coupling between the Au core and sulfide shell, and therefore, Au@ZnS–AgAuS Y–S NCs are expected to be effective visible light pholocatalysts.

**Figure 4:**
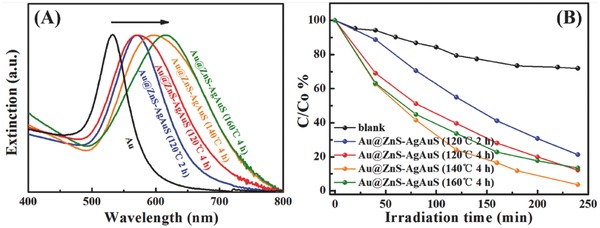
A) UV–vis extinction spectra of Au NCs and as‐prepared Au@ZnS–AgAuS Y–S NCs. B) Photodegradation curve of methyl blue (MB).

To investigate the photocatalytic activities of the as‐synthesized samples, the photodegradation of methyl blue (MB) was carried out in visible light region, as shown in Figure 4B. The four products all show photodegradation ability to MB and Au@ZnS–AgAuS (140 °C 4 h) Y–S NCs exhibit the highest efficiency. The plasmon‐extinction coupling between Au core and semiconductor shell changes along with the evolution of Au core size, shape, and semiconductor shell composition. In the case of Au@ZnS–AgAuS (140 °C 4 h) Y–S NCs, the optimization of plasmon‐exciton coupling together with ZnS–AgAuS grain boundary and Au cocatalyst coordination lead to their higher photodegradation activity.

Hydrothermal cation exchange achieved Au@ZnS–AgAuS (140 °C 4 h) Y–S NCs are selected for the photocatalytic H_2_ evolution activity measurement under visible light irradiation (λ > 420 nm), and aqueous cation exchange in the air enabled Au@ZnS core‐shell NCs are chose as comparison (Figure S8, Supporting Information). As shown in **Figure** **5**, Au@ZnS core‐shell NCs exhibit bad H_2_‐production activity under visible light region, whereas Au@ZnS–AgAuS (140 °C 4 h) Y–S NCs show a high hydrogen production rate of 880 µmol h^−1^ g^−1^.

**Figure 5:**
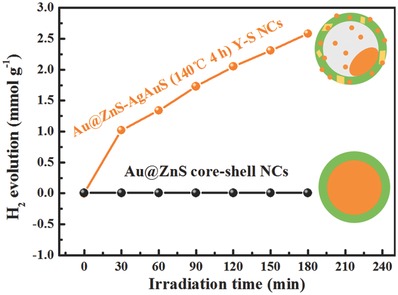
Visible light photocatalytic H_2_‐production of Au@ZnS–AgAuS (140 °C 4 h) Y–S NCs, Au@ZnS core‐shell NCs.

It has been reported that pure ZnS exhibits negligible photocatalytic activity in the visible light region. In the case of Au@ZnS core‐shell NCs, visible light irradiation (*hν* ≈ 2.24 eV) induces localized surface plasmon generation in the Au core. Owing to the high conduction band position (−1.75 eV vs normal hydrogen electrode (NHE)) of ZnS, the photogenerated electrons in the SPR state of Au core cannot inject into the conduction band of ZnS, resulting in the negative visible light photocatalytic activity.^28^ As for Au@ZnS–AgAuS Y–S NCs, the cation exchange reaction enabled ZnS–AgAuS heterointerfaces and possible cation (such as Ag^+^, Au^+^) doping of ZnS (which is under further study) may reduce its conduction band position, thus enable the efficient plasmon‐induced eletron/hole separation and maybe hot electrons injection to ZnS shell, then transit in the grain boundaries between narrow band gap semiconductor AgAuS (0.97 eV). By this means, the visible light response and reduced electron–hole recombination rate can be achieved. As theoretically described by Mie theory, the LSPR intensity and wavelength dependents on the particle size, shape, and dielectric constant of the surrounding medium. When there exist Au cocatalysts (≈2–4 nm) coordination, the LSPR effect is very weak, and the better electron affinity enables the Au cocatalysts to collect the chemical‐useful‐carrier, thus further boosting the photocatalytic efficiency.^29^

In summary, we have developed a hydrothermal cation exchange strategy to fabricate Au@ZnS–AgAuS Y–S NCs with well‐controlled void evolution, metal (Au, Ag) ion doped ZnS, programmed ZnS–AuAgS heterograin boundaries and Au cocatalyst combination. The formation mechanism can be reasonably understood by the cation exchange initialized chemical etching and diffusion of Au core through the sulfide shell. The fabrication of the several distinct structures achieves the optimization of plasmon‐exciton coupling and plasmon enhanced electron–hole separation, endowing the Y–S NCs with high photocatalytic activities under visible light irradiation. The concept of this strategy can be useful for the synthesis of other noble metal and widespread semiconductor hybrid composites with tunable Y–S structures and new applications.

## Experimental Section

*Chemical and Reagents*: Gold (III) chloride trihydate (HAuCl_4_•4H_2_O, 99.9+%), silver nitrate (AgNO_3_, 99+%) were all purchased from Aladdin Reagent, and other reagents are reagents grade and purchased from Beijing chemical factors. All of reagents were used as received without further purification.

*Synthesis of 35 nm Au NCs and Au@Ag Core‐Shell NCs*: Au nanoparticles with diameter of 35 nm were used. A seed‐mediated approach was utilized to synthesize Au NCs. To be specific, a seed solution was prepared by mixing 2.5 mL of 0.15 m CTAB and 1.25 mL 0.001 m HAuCl_4_ aqueous solution in a 50 mL plastic tube. Then 0.3 mL, 0.01 m freshly prepared, ice‐cold NaBH_4_ was added into the mixture under vigorous magnetic stir for 2 min and then was transferred to a 30 °C water bath aging for 1 h. And brown Au seeds were obtained. For the growth solution, 3.2 mL 0.2 m CTAB, 0.8 mL, 0.01 m HAuCl_4_ and 3.8 mL 0.1 m ascorbic acid were added into 35 mL ultrapure water and mixed uniformly. Then 40 µL seed solution was added to the growth solution and aged at room temperature for at least 7 h. 30 mL of Au colloid was centrifugation at 7000 rpm for 10 min and redispersed in 10 mL 25 × 10^−3^ m CTAB aqueous solution. Subsequently, ascorbic acid (0.7 mL 0.1 m), AgNO_3_ (0.3 mL 0.01 m) and NaOH (1 mL 0.1 m) was added in order and stirred uniformly. An orange Au@Ag colloidal suspension forms after aging at 30 °C for 1 h.

*Preparation of Sulfur Precursor*: In brief, 64 mg of sulfur powder was mixed with 10 mL oleic acid and 5 mL oleylamine in a 50 mL round‐bottom flask followed by transferring it to an oil bath and magnetic stirred at 100 °C for 1 h. When the solution was cooled down to room temperature, 15 mL toluene was added then mixed together and the S precursor was obtained.

*Hydrothermal Synthesis of Au@ZnS–AgAuS Y–S NCs*: For the preparation of Au@AgAuS core‐shell NCs, 100 µL as‐prepared S precursor was add to the Au@Ag aqueous suspension followed by aging at 30 °C for 1 h. The Au@AgAuS nanoparticles were separated from the solution by centrifugation (7000 rpm 10 min) and finally redispersed in 10 mL of CTAB (50 × 10^−3^ m). For the preparation of Au@ZnS–AgAuS Y–S NCs, 2 mL of 0.1 g mL^−1^ Zn(NO_3_)_2_ aqueous solution and 50 µL TBP was added to the aqueous Au@AgAuS suspension and then transferred into a 50 mL volume autoclave. By controlling the hydrothermal experimental condition to be 120 °C for 2 h, 120 °C for 4 h, 140 °C for 4 h, and 160 °C for 4 h, we obtained Au@ZnS–AgAuS (120 °C 2 h), Au@ZnS–AgAuS (120 °C 4 h), Au@ZnS–AgAuS (140 °C 4 h), Au@ZnS–AgAuS (160 °C 4 h) Y–S NCs, respectively.

*Synthesis of Au@ZnS Core‐Shell NCs in the Air*: The process of Au@ZnS core‐shell NCs synthesis was similar to that of Au@ZnS–AgAuS Y–S NCs. The fabrication procedures were basically the same, except for the cation exchange reaction was carried out at 60 °C for 2 h in a water bath instead of hydrothermal treatment.

*Characterizations*: The crystalline structures of Au@ZnS–AgAuS Y–S NCs were characterized by Powder X‐ray Diffraction measurements using a Bruker D8 multiply crystals X‐ray diffratometer (10° per min). LRTEM images were obtained by HITACHI H‐7650 electron microscopy operating at 80 kV. HRTEM images and EDS were obtained using transmission electron microscopy (FEI Tecnai G2 F20 S‐Twinworking at 200 kV) equipped with X‐ray energy‐dispersive spectroscopy detector. UV‐Vis spectra of samples were recorded on a Shimadzu UV3600 UV‐Vis spectrophotometer at room temperature (RT).

*Photocatalytic Degradation Test*: MB was selected as the model organic compound to study the photocatalytic activity of the as‐prepared products. In a typical photocatalytic experiment, 1 mg of catalyst was dispersed by a constant stirring in mixed aqueous solution containing 4 mL ultrapure water and 16 µL of 0.5 mg mL^−1^ MB. Before irradiation, the mixture was magnetically stirred for 30 min in the dark to achieve the adsorption and desorption equilibrium between them. 300 W Xe lamp with a 400 nm cut‐off filter (CEL‐HXF300/CEL‐HXUV300) was used as the light source to irradiate the suspension. The supernatant was obtained by centrifugation at a speed of 7000 rpm for 10 min at a given time and followed by measuring by the UV–vis absorption spectrometer.

*Photocatalytic H_2_ Production Activity test*: The photocatalytic hydrogen production experiments were carried out in a 250 mL multiport flask at ambient temperature and atmospheric pressure, and outlets of the flask were sealed with silicone rubber septum. A 300 W Xe lamp with application of a 420 nm cut‐off long pass filter was used as a light source to trigger the photocatalytic reaction. The water splitting reaction rate was monitored by measuring the evolved gases using gas chromatography (GC7890, Agilent Technology).

## Conflict of Interest

The authors declare no conflict of interest.

## Supporting information

Supplementary
