# Supplementary material for: Hydrothermal Cation Exchange Enabled Gradual Evolution of Au@ZnS–AgAuS Yolk–Shell Nanocrystals and Their Visible Light Photocatalytic Applications
Source: Adv Sci (Weinh). 2017 Nov 20;5(1):1700376. doi: 10.1002/advs.201700376 (PMC5770678; doi:10.1002/advs.201700376)
Supplement: Supplementary file 1 — Supplementary [file ADVS-5-na-s001.pdf]

Copyright WILEY-VCH Verlag GmbH & Co. KGaA, 69469 Weinheim, Germany, 2017.

ADVANCED SCIENCE
Open Access

Supporting Information

for *Adv. Sci.,* DOI: 10.1002/advs.201700376

Hydrothermal Cation Exchange Enabled Gradual Evolution of Au@ZnS–AgAuS Yolk–Shell Nanocrystals and Their Visible Light Photocatalytic Applications

*Jingwen Feng, Jia Liu, Xiaoyan Cheng, Jiajia Liu, Meng Xu, and Jiatao Zhang**

Copyright WILEY-VCH Verlag GmbH & Co. KGaA, 69469 Weinheim, Germany, 2016.

Supporting Information

Hydrothermal Cation Exchange Enabled Gradual Evolution of Au@ZnS-AgAuS Yolk-shell Nanocrystals and Their Visible Light Photocatalytic Applications

*Jingwen Feng, Jia Liu, Xiaoyan Cheng, Jiajia Liu, Meng Xu, Jiatao Zhang**

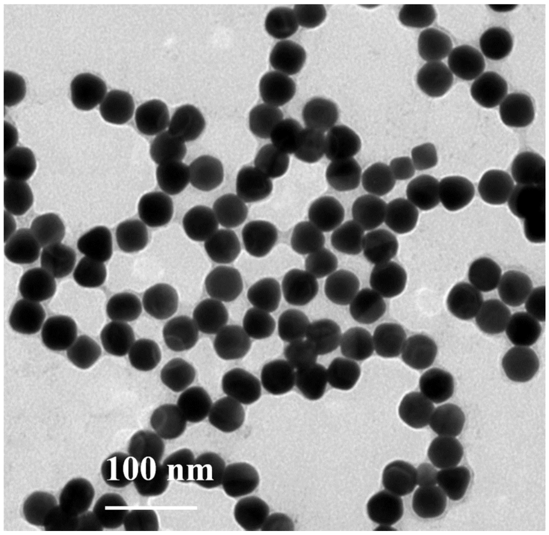

Figure S1 TEM image of Au core.

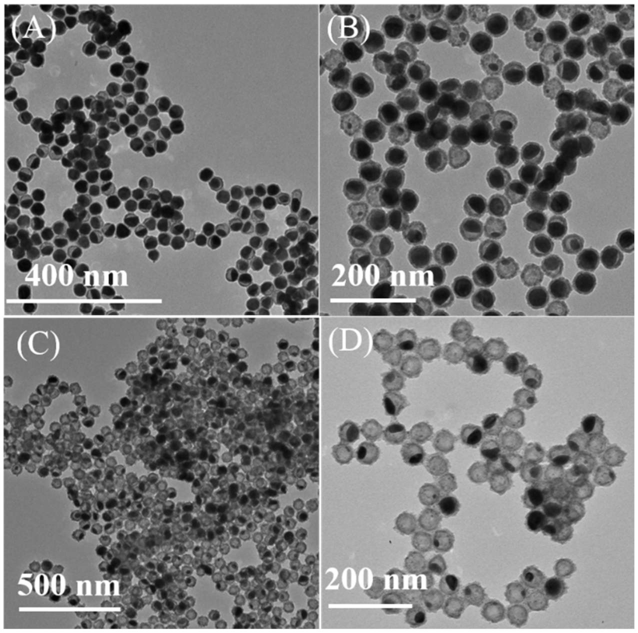

Figure S2 Large-scale TEM images of A) Au@ZnS-AgAuS (120 °C 2 h), B) Au@ZnS-AgAuS (120 °C 4 h), C) Au@ZnS-AgAuS (140℃ 4 h) and D) Au@ZnS-AgAuS (160 °C 4 h)Y-S NCs

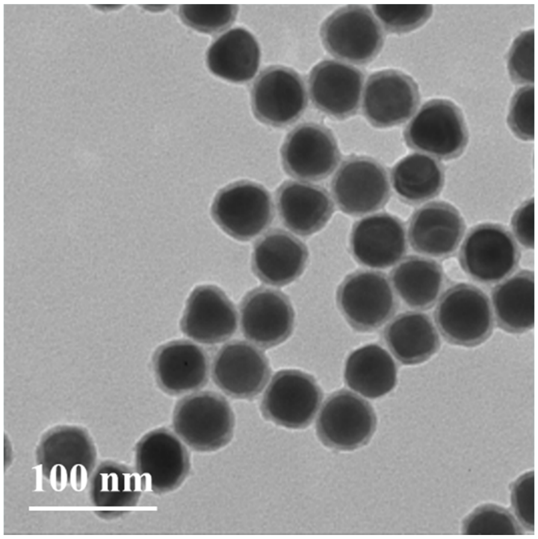

Figure S3 TEM image of Au@ZnS-AgAuS NCs, which were obtained at 120 °C for 30 min.

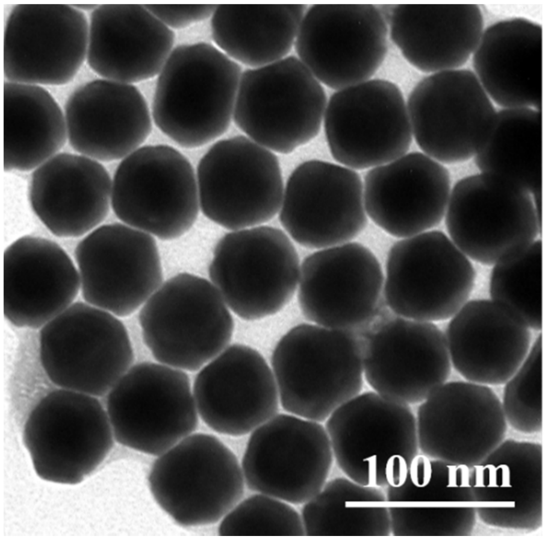

Figure S4 TEM image of Au@AgAuS, obtained at 140 °C for 4 h. (absence of Zn^{2+} and TBP)

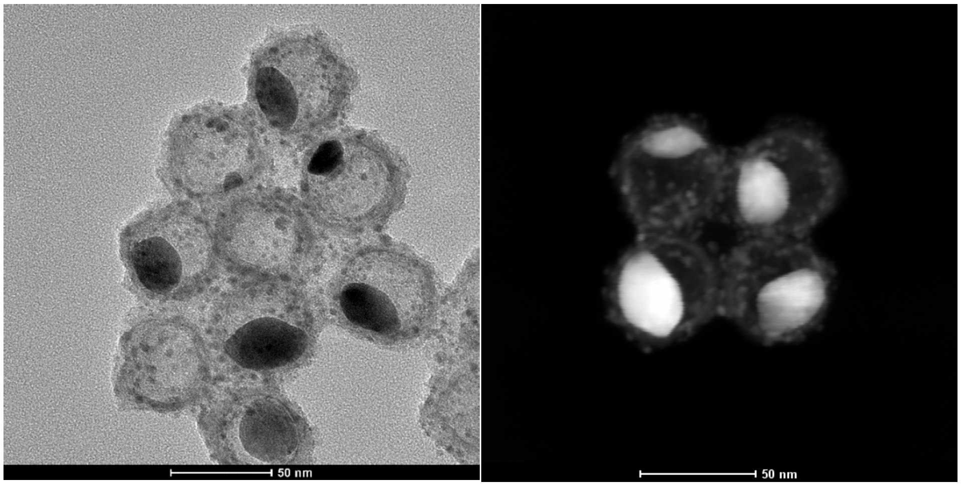

Figure S5 TEM and STEM images of Au@ZnS-AgAuS (140 °C 4 h) Y-S NCs.

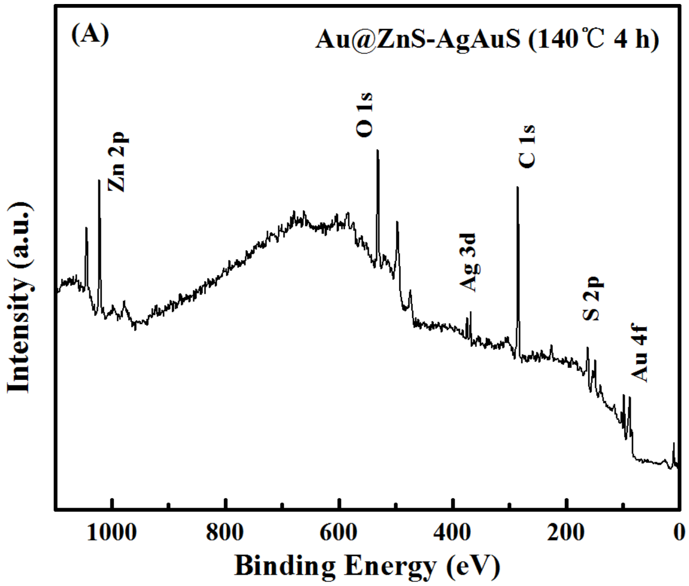

Figure S6 XPS spectra of Au@ZnS-AgAuS (140 °C 4 h) Y-S NCs

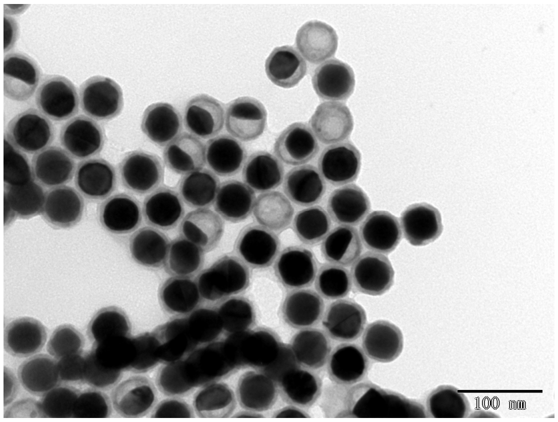

Figure S7 TEM image of Au@ZnS-AgAuS Y-S NCs in the presence of CTAC. (150 ℃ for 4 h)

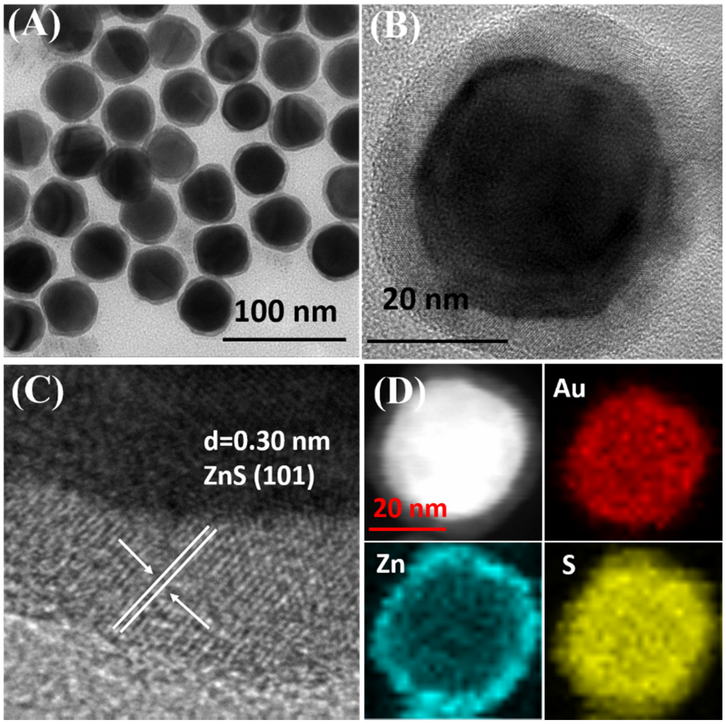

Figure S8 A, B) TEM image and of Au@ZnS core-shell NCs. C) HRTEM image D) STEM-EDS elemental maps of Au@ZnS core-shell NCs.
